# Development of flip-chip technology for the optical drive of superconducting circuits

**DOI:** 10.12688/openreseurope.17481.1

**Published:** 2024-04-29

**Authors:** Oliver Kieler, Hao Tian, Marco Kraus, Shekhar Priyadarshi, Judith Felgner, Alexander Fernandez Scarioni, Johannes Kohlmann, Mark Bieler

**Affiliations:** 1 Quantum Electronics, Physikalisch-Technische Bundesanstalt, Braunschweig, Lower Saxony, 38116, Germany

**Keywords:** photodiode, gold stud bumps, flip chip technology, superconducting quantum circuits, cryo-electronics, AC Josephson Voltage Standard, JAWS

## Abstract

We discuss the flip-chip mounting process of photodiodes and fiber sleeves on silicon substrates to meet the increasing demand for fabrication of highly integrated and hybrid quantum circuits for operation at cryogenic temperatures. To further increase the yield and success rate of the flip-chip procedure, the size of the gold stud bumps, and flip-chip parameters were optimized. Moreover, to connect optical fibers to the photodiodes in an optimal position, the fiber sleeves were aligned with specially fabricated alignment circles before applying thermocompression with the flip-chip machine. The mounted photodiodes were tested at both room temperature and cryogenic temperature, and we find that mechanical imperfections of the sleeve-ferrule combination limit the overall alignment accuracy. The experimental results show that our flip-chip process is very reliable and promising for various optical and electrical applications and, thus, paves the way for fabrication of hybrid chips, multi-chip modules and chip-on-chip solutions, which are operated at cryogenic temperatures.

## Plain Language Summary

The paper summarizes the efforts to establish a flip-chip technology at PTB for realization of multi-chip modules, especially for the optical pulse-drive of the pulse-driven AC Josephson Voltage Standard (also called: Josephson Arbitrary Waveform Synthesizer - JAWS), having a large impact in electrical quantum metrology.

Motivation: For the application in JAWS, several high-speed photodiodes (PDs) will be mounted on a Si carrier chip by flip-chip (thermo-compression). This carrier chip is patterned with suitable electrical HF circuits to transmit the GHz electrical pulses (generated by the photodiodes) to the JAWS chip, which is next to this carrier chip.

The PDs are backside illuminated, i.e. there is a lens on the backside to introduce the optical pulses. To attach and align the optical fibers (one fiber to each PD), glass sleeves are mounted by the flip-chip machine using a customized tooltip. These sleeves are glued to the Si carrier chip.

The paper is structured as followed:

After a short introduction in chapter I, the next chapter II describes our efforts to optimize the Au stud-bumps to increase the yield of the flip-chip.

The main chapter III describes the optimization of both packaging steps:

Mounting of the PDs

Mounting of the sleeves.

This includes all flip-chip parameters, test chips for checking the electrical yield, thermal-cycling (300 K @ 4 K) and estimation of the achieved alignment precision.

The chapter IV showed some measurements under CW laser irradiation of the alignment precision of the PDs relative to the sleeves’ position and the rotation angle of two different optical fiber ferrules.

A yield of 95% for more than 250 PDs mounted by this method was achieved, which is more than enough for our purpose.

## 1. Introduction

The so-called flip-chip technology is a packaging technique used mainly in the semiconductor industry to connect semiconductor devices, such as integrated circuits (ICs), to external circuitry. This technology was firstly developed by IBM in the 1960s ^1^. It is basically a bonding technique, where the electrical connections are made between the chip and its packaging substrate by inverting the chip facing down onto the packaging substrate. By using conductive metal bumps, the bonding pads of the chip have an electrical and mechanical connection with the bond pads of the packaging substrate.

A typical flip-chip process consists of the following steps:

1. Creating stud bumps on the active side of the fabricated chip or the substrate.

2. The substrate is fabricated with an array of pads matching to the chip bumps. (Of course, the solder bumps might also be fabricated on the substrate, with matching pad arrays on the chip.)

3. Chip and substrate are aligned with precision to ensure accurate positioning of the stud bumps and pads.

4. The chip is flipped, and the bumps are brought into contact with the substrate pads.

5. The assembly is heated to a temperature at which the stud bumps melt and reflow while a controlled force is applied, creating a secure and reliable electrical connection.

6. Optional underfill material may be applied between the chip and the substrate to enhance the mechanical strength and to provide additional protection.

7. The flip-chip assembly undergoes a testing procedure to ensure proper functionality.

The flip-chip technology has several advantages over the standard wire bonding technique. Firstly, it reduces the electrical interconnection length, and the signal propagation delays. Reduced inductance and capacitance in the interconnects lead to improved electrical performance. For example, the inductance of a bond wire with a diameter of 25 µm is about 1 nH per mm length ^2^. Thus, flip-chip technology is well-suited for applications requiring high-frequency performance, such as in radio frequency (RF) devices. Moreover, the compact nature of flip-chip assemblies enables higher packaging density, making it suitable for high-performance applications. The direct connection of the chip die to the substrate enhances thermal conductivity, improving heat dissipation. Effective thermal management is crucial to prevent overheating, especially in high-power applications. Despite these advantages, the flip-chip process is more complex than wire bonding, requiring precision alignment and careful control of the soldering process. The reliability of flip-chip assemblies is influenced by factors such as solder joint integrity and underfill material properties. If the flip-chip assembly is aimed for operation at cryogenic temperatures, the challenges further increase, as the thermal expansion of the different materials must be considered. The adoption of flip-chip technology not only depends on the aforementioned factors but also on cost considerations, the complexity of the application, and the required reliability standards ^3^.

In the field of quantum metrology, the so-called Josephson Arbitrary Waveform Generator (JAWS) ^4– 6^ has played a very important role. The JAWS is basically a quantum accurate digital-to-analogue converter. Here, a series array of SNS (S…superconductor, N…normal conductor) Josephson junctions is driven by a train of short current pulses with a typical maximum pulse repetition frequency of 15 GHz. Each pulse transfers a flux-quanta through each Josephson junction resulting in a quantized output waveform. National Metrology Institutes as NIST (National Institute of Standards and Technology, US) and PTB (Physikalisch-Technische Bundesanstalt) have been working on JAWS for many years. JAWS circuits from NIST or PTB are nowadays in use at several Metrology Institutes for metrology applications and further investigations. One of the approaches to further develop the JAWS experimental set-up is to implement modules for optical-pulse drive. This constitutes a substitution and possible improvement for the electrical pulse-driven method realized by pulse pattern generators (PPG) ^4, 6^. Optical pulse drive not only reduces the cost of the JAWS set-up, but it also has the potential to increase the repetition rate of the input pulses, which, in turn, will increase the synthesized output voltage and frequency. For this purpose, in previous studies ^7, 8^, high-speed photodiodes (PD) were mounted by flip-chip technology onto the silicon chips and integrated in the JAWS system. In this case, the PDs are placed at cryogenic temperatures and convert the optical pulses transmitted via optical fibers from room temperature to electrical pulses, which drive the JAWS circuits. According to the recent first measurement results ^8– 10^, bipolar quantized sinusoidal waveforms were successfully synthesized at 4 K by using one or two PDs that were flip-chip mounted to a silicon substrate.

This paper describes the establishment of the flip-chip procedure at PTB in detail, including the optimization of stud bumps ( Section 2), the optimization of the mounting process of the PDs by flip-chip and sleeves by gluing ( Section 3), and eventually the characterization of the mounted PDs ( Section 4).

## 2. Optimization of stud bumps

Before starting the mounting of the PDs by the flip-chip process, gold stud bumps have to be attached onto the gold bond pads of the PD carrier chip (silicon or GaAs substrate) by using a commercial wire bonder system (TPT HP 10). The size of these gold stud bumps is quite critical, not only because the PDs to be mounted (Albis PD20X1-L2Q) are very compact (size: 350 µm × 350 µm), but also the width of the signal line and the gaps of our coplanar waveguide (CPW), that guides the current pulses from the PD to the superconducting circuit are 73 µm and 30 µm, respectively. Thus, the diameter of the stud bumps needs to be kept small and uniform to avoid electrical shorts between the signal and the ground conductors. To obtain a smaller ball size, the bonding parameters (tail length, temperature, force, etc.) of the ball bonder machine were firstly optimized in the semi-auto mode.

**Figure 1.**
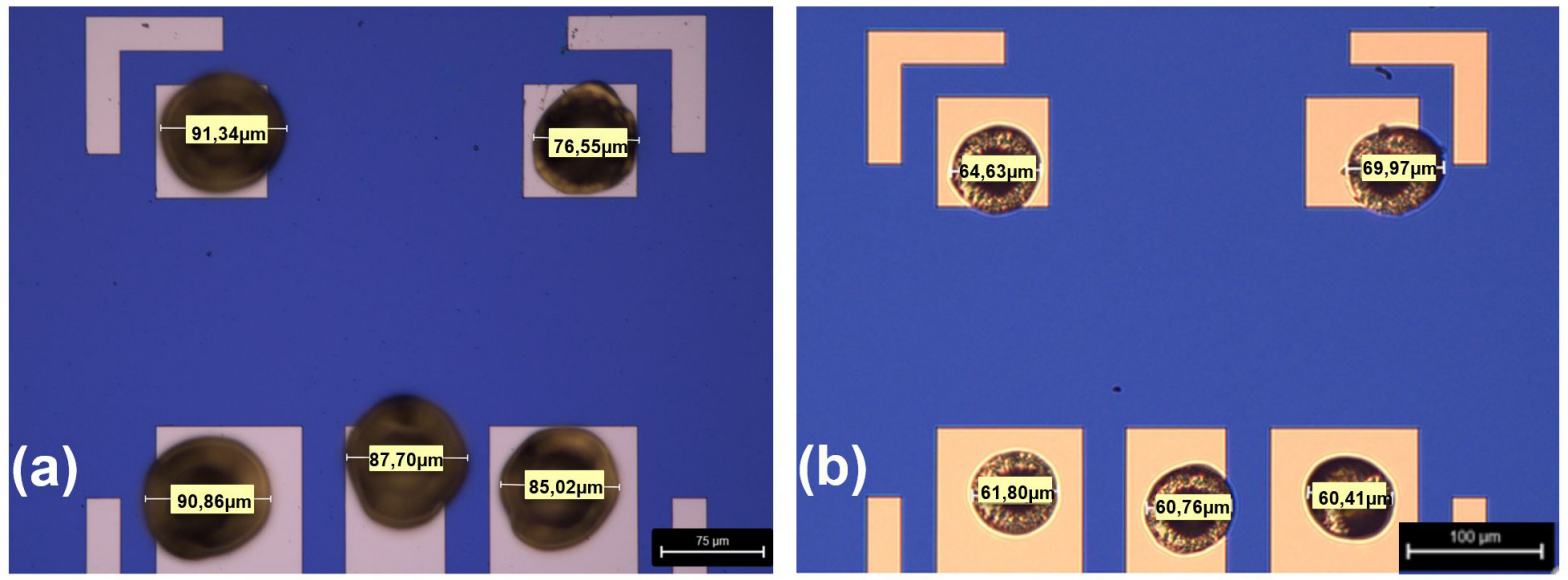
Typical microscope image of the size of the gold stud bumps ( **a**) before optimization (25 µm diameter of the gold wire) and ( **b**) after optimization (17 µm diameter of the gold wire). The dimension of each ball is labeled.

In addition to the bond parameters, the ball size also depends on the diameter of the wire used in the bonding process. The initially used gold wire had a diameter of 25 µm, which produced comparatively large stud bumps, because the ball size will never be smaller than approximately three times the wire size. This often led to long tails and short circuits between the narrow CPW lines. For this reason, the wide wire was replaced with a thinner gold wire (diameter of 17 µm). As shown in Figure 1 (as a typical example), the diameter of the gold stud bumps was significantly reduced after the wire exchange and parameter optimization, yielding a reduction of the size of the stud bumps around 25%. Due to the smaller size of the stud bumps, the risk of short circuits during the flip chip process was avoided completely. A mean size of the bumps of (63.8 ± 3.4) µm was determined by analyzing 60 Au-bumps. This average bump size is slightly smaller than described in 11 65…80 µm. By applying the following bond parameters, a very good yield of the stud bumps of nearly 100% was obtained: ultra-sonic power 100 mW, Electronic Flame-Off (EFO) power 90%, bond-time 150 ms, bond-power 12 cN, chuck-temperature 40°C, tool-temperature 160°C. In contrast to 11, 12 “long” tails (causing shorts) could be avoided completely too.

## 3. Flip-chip procedure of PDs and mounting of sleeves

After optimization of the stud bumps, the photodiodes were flip-chip mounted using the flip-chip machine (Finetech Fineplacer Sigma). Several custom-designed and changeable tooltips are available, which are suited for different chip sizes and geometries. Firstly, the miniature PD was picked up by the tool tip in face-down configuration. Afterwards, the gold pads of the PD and the gold stud bumps on the carrier chip were aligned by using two overlaid microscope images of the flip-chip machine. A thermocompression flip-chip process was then applied. In the thermocompression bonding, usually two metals (in our case gold) are joined by applying a constant force at a raising temperature. To achieve less bump deformation, a bonding force of 4 N or 5 N ^11– 13^ for the 5 pads (each with one bump) was used. During this process, the temperature gradually increased up to 250°C within a time of 5 s, stayed at 250°C for about 60 s and then reduced to 100°C within a time of 150 s. No underfill glue was needed for this process.

**Figure 2.**
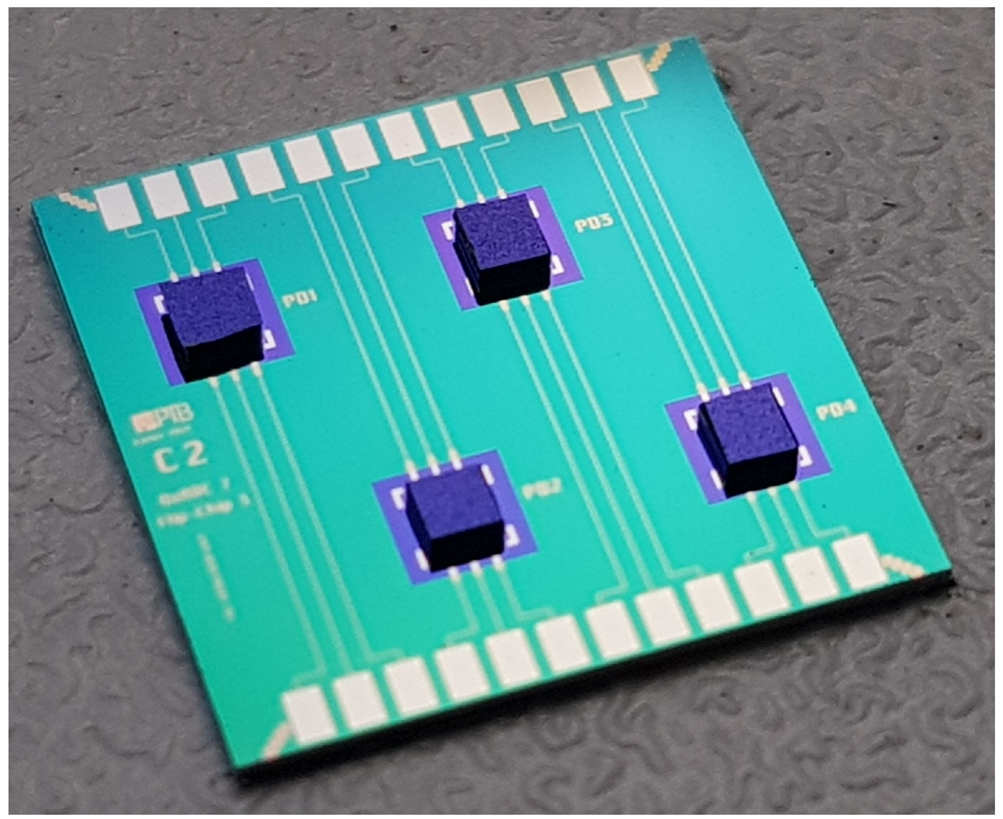
Optical photograph of four dummy chips (size: 350 µm × 350 µm) successfully mounted on the silicon substrate using flip-chip technology.

To optimize the flip-chip parameters (bonding force, force/temperature ramp, bump alignment, bump tails) and to estimate the yield of the mounted chips, on-chip test structures were fabricated. The silicon substrate (shown in Figure 2) has four identical simple circuits each consisting of three extended gold lines. The dummy chips, which simply connect the interrupted gold lines of the substrate with each other, were mounted on the test substrate using flip-chip technology. These mounted chips were on-chip electrically characterized (DC measurements) by a wafer prober at 300 K and in a liquid Helium dewar at 4 K. The main purpose for this measurement was to check the electrical connections resulting from the flip-chip process, especially after cooling down to 4 K. The measured results showed that all 4 mounted dummy chips behave as expected (yield 100%, see next section for the yield for mounting many PDs). All opposite lines are connected via the dummy chip, no short circuit between two adjacent lines could be detected, and no degradation was observed after repeated thermal cycling between 300 K and 4 K.

**Figure 3.**
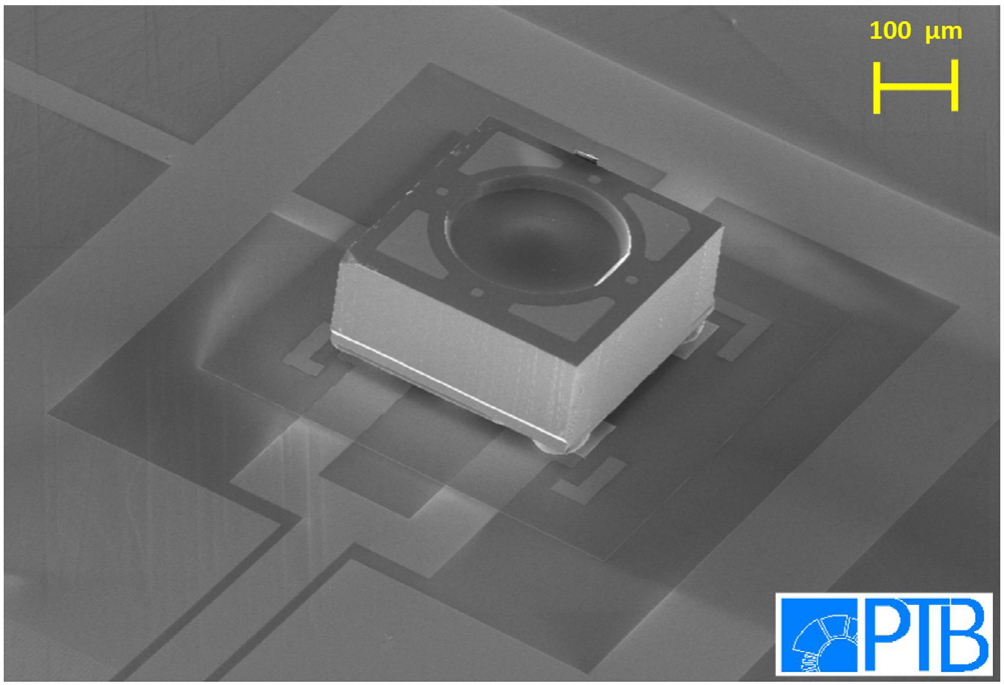
Tilted SEM image of a PD mounted onto a high-frequency line on a silicon substrate. The integrated lens on the backside of the PD is clearly visible.

After successfully mounting and testing the electrical connections of the dummy chips, we have started to mount real PDs using the aforementioned technique. Since then, many PDs were mounted onto different chip substrates. We also investigated our mounted chips under a Scanning Electron Microscope (SEM). The SEM image of Figure 3 displays that the mounted PDs were attached nicely to the gold stud bumps underneath. No tilt or twist of the PDs was observed. After optimization of the flip-chip procedure we achieved a yield better than 95% for a total of more than 250 mounted PDs. So far, no PD has been destroyed during or after the flip chip procedure. No PD got detached due to repeated thermal cycling between 300 K and 4 K. Short circuit of the PDs occurred only occasionally due to the unideal placement of the gold stud bumps. This high yield is required due to the relatively high costs of the high-performance PDs and shows that the established flip-chip technology is very robust and reliable.

The employed PDs need to be illuminated from their backside and have an additional integrated lens in their substrate. To connect the optical fiber to the backside-lensed PDs, special fiber sleeves need to be mounted and glued on the carrier chip as precisely as possible. To align the sleeves under the microscope, alignment circles were fabricated on the chip. In the chip designs, the inner diameter of the circles, which are electrically passive, were perfectly matched to the outer diameter of the sleeves.

For flip-chip bonding of the sleeves a specially designed and fabricated sleeve tooltip was installed to the flip-chip machine. The sleeve with a length of 7 mm was picked up by the tooltip and was aligned to the alignment circle by using the two overlaid microscope images of the flip-chip machine. Then a small amount of Stycast 12661 epoxy glue (mixing ratio of Part A to Part B was approximately 1 to 0.28) was applied carefully on the chip substrate. Afterwards, the mounting step started, and the sleeve was carefully lowered by the tooltip and placed on the alignment circle of the substrate. Lastly, to harden the glue, the mounted chips were cured at room temperature for 24 hours and then baked at 80°C for 2 hours, as recommended in the datasheet of the glue.

**Figure 4.**
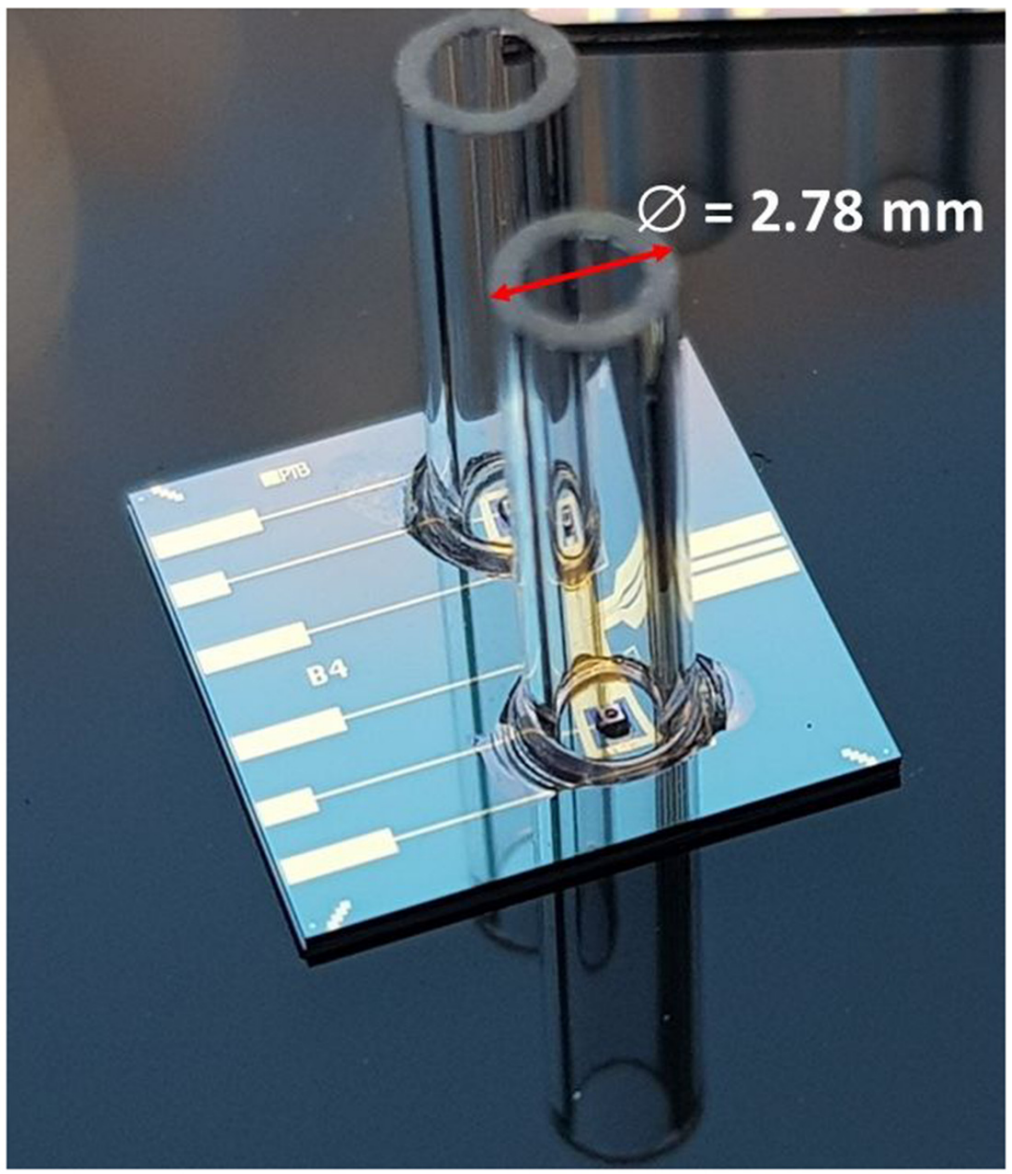
Optical photograph of two sleeves mounted using flip-chip technology and glued to the silicon substrate. The sleeves have a length of 7 mm.

**Figure 5.**
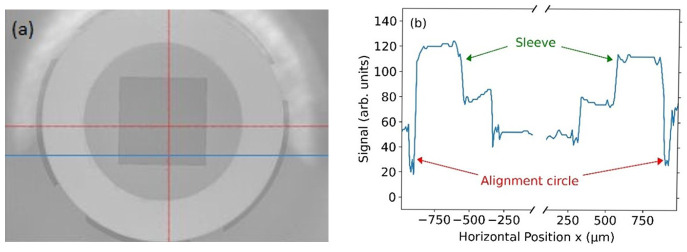
( **a**) Microscope image of the sleeve attached to the silicon substrate. On the substrate alignment circles have been fabricated at the positions corresponding to the outer diameter of the sleeve. The red cross just marks the center of the camera image. ( **b**) Intensity image values in arbitrary units along the blue line in ( **a**). The alignment circles with a nominal width of 50 µm can be seen at the left- and right-hand side of the plot. The measured width of both alignment circles differs by less than 15 µm, which denotes the alignment accuracy of the sleeve and thus of the fiber-chip coupling.

Figure 4 shows an optical photograph of two sleeves being glued onto the chip substrate using the aforementioned process. In order to determine the alignment accuracy, we analyzed several microscope images, as shown in Figure 5(a). An intensity analysis of the images (see Figure 5b) allowed us to quantitatively determine this alignment accuracy to be better than 15 µm. At first this number seems to be too large for single-mode fibers with a core diameter below 10 µm. Yet, this accuracy is more than sufficient for our applications, because the active area of the backside lens of the PD has a diameter of 100 µm. To test the quality of the glued sleeves, the chip was repeatedly cooled down and warmed up between 4 K and 300 K. After five cooling-down and warming-up cycles, the mounted sleeves remained stable and stuck firmly onto the chip.

## 4. Characterization of the mounted PDs

**Figure 6.**
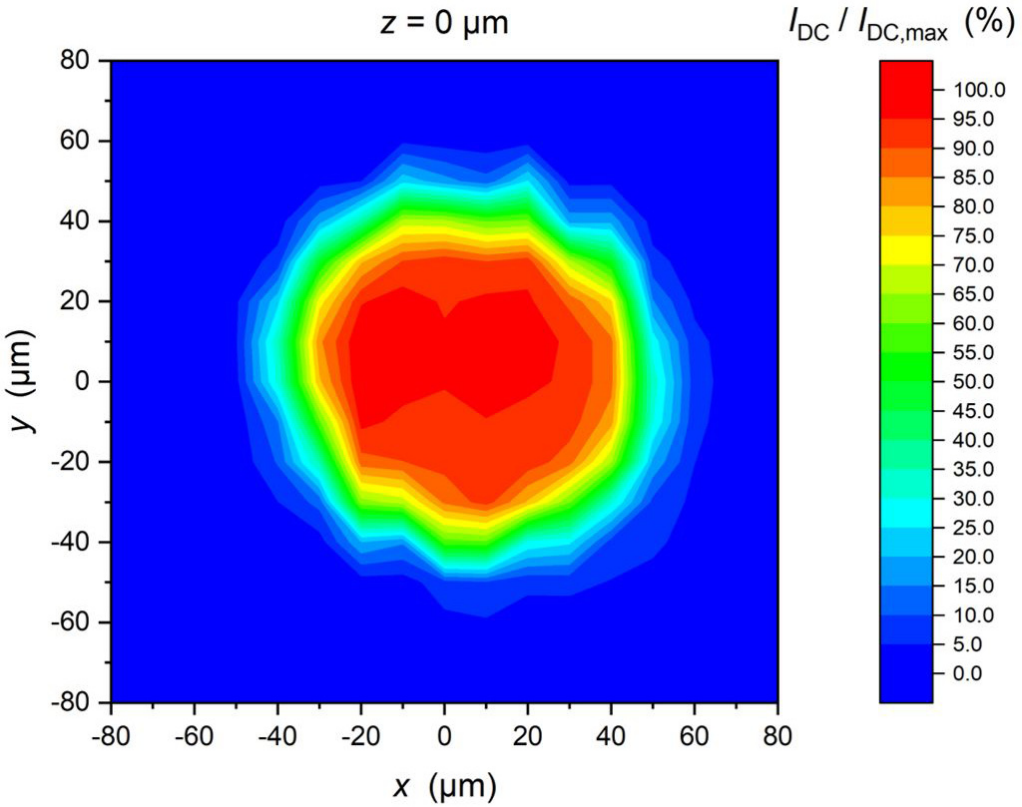
Dependence of the photocurrent of one PD under irradiation by CW laser light with a power of 1.6 mW versus x-y position of the ferrule over the PD. The measured photocurrent was normalized to I _dc (max)_ = 1.2 mA. The distance of the ferrule to the PD was z = 0 µm.

The mounted PDs with and without the sleeves were investigated under Continuous Wave (CW) laser light at room temperature. The CW laser light had a wavelength of 1310 nm, power of 1.6 mW and it was guided onto the backside of the PDs via an optical fiber, which ends in a pigtail ferrule (the glass body of the ferrule was placed directly above the PD). In this case, no sleeve was mounted on the chip. With the help of a positioning table, the chip was moved relative to the ferrule with a step size of 10 µm in the x/y/z direction. Depending on the PD position, the photocurrent of the photodiode was measured and is a measure of the optical coupling. The applied reverse bias of the PD was 4 V. In Figure 6, the measured results show a nice Gaussian like shape with a full width at half maximum of 85 µm. This corresponds very well to the 100 µm diameter of the built-in lens of the PD.

**Figure 7.**
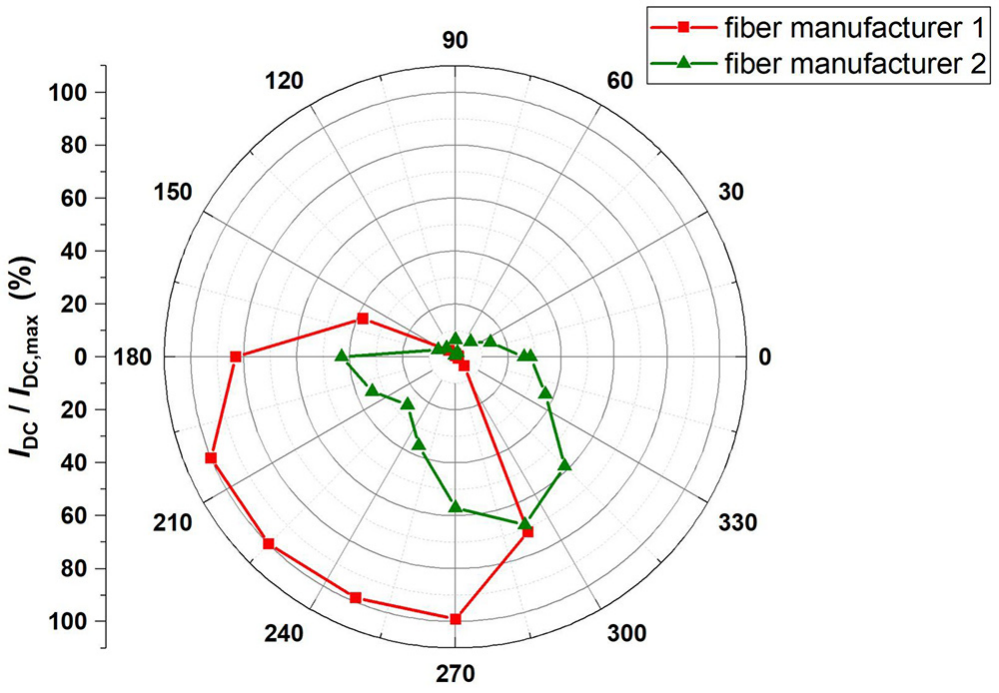
Dependence of the DC output current of one PD under irradiation by CW laser light with a power of 1.6 mW versus rotation angle of the chip with respect to the ferrule. The measured results were normalized to I _dc (max)_ = 1.2 mA. Two different types of optical fibers were used. They both exhibit a similar behavior.

Next, the mounted PDs together with the glued glass sleeves and attached ferrules were characterized as well. To this end we like to note that we used normal ferrules, without any angles facet. As previously mentioned, we have not yet achieved a perfect alignment between the center of the PD and the glass sleeve, with inaccuracies below 15 µm. However, as shown in Figure 7, the amplitude of the photocurrent is largely dependent on the rotation angle of the chip with respect to the ferrule and varies between a maximum value and zero. When only considering the accuracy of 15 µm and the built-in lens of the PD with a diameter of 100 µm, we would not expect such a considerable change of the photocurrent. Thus, it is clear that mechanical imperfections of the ferrule-sleeve combination limit the overall alignment accuracy. Two optical fiber-ferrule combinations were tested in the measurement as shown in Figure 6 and studies on other sleeves and ferrules confirm this finding. Despite these mechanical imperfections, maximum optical coupling can be obtained by adjusting the rotation angle of the chip and/or the fiber at room temperature and keep it fixed during operation at 4 K. We will perform further investigations on ferrule and sleeves products from different manufacturers to investigate this issue in more detail.

## 5. Conclusions

To summarize our work, we successfully established the flip-chip procedure of mounting PDs and glass sleeves at PTB. Our method and statistical analysis support and adds new information to our and our partners previous publications obtained in the framework of different joint projects ^7, 10– 13^. After mounting more than 250 PDs with an overall yield better than 95%, this technique has proven to be very reliable and reproducible for our purpose of realizing an multi-channel optical pulse-drive of JAWS chips at low temperatures. We also studied the alignment accuracy of glass sleeves and ferrules. While the sleeves can be mounted to substrates with an accuracy better than 15 µm, the overall alignment accuracy of ferrule-sleeves combinations is mainly determined by mechanical imperfections of the two components. In the future, the flip-chip technology will be used at PTB for different applications ranging from optical measurement to quantum technologies.

## Ethics and consent

Ethical approval and consent were not required.

## Disclaimer

Commercial equipment is identified in this paper to adequately specify the experimental procedure. Such identification does not imply recommendation or endorsement by PTB.

## Data Availability

Open Access Repository of the Physikalisch-Technische Bundesanstalt: Development of Flip-Chip Technology for the Optical Drive of Superconducting Circuits. https://doi.org/10.7795/720.20240226 ^14^. Data are available under the terms of the Creative Commons Attribution 4.0 International license (CC-BY 4.0).
