# Supplementary material for: Development of flip-chip technology for the optical drive of superconducting circuits
Source: Open Res Eur. 2024 Nov 11;4:97. Originally published 2024 Apr 29. [Version 2] doi: 10.12688/openreseurope.17481.2 (PMC11585851; doi:10.12688/openreseurope.17481.2)
Supplement: Peer review history

# Reviewer report: Reviewer response for version 2

**DOI:** 10.21956/openreseurope.20301.r46241

**Published:** 2024-12-05

**Authors:** Ming-Han Chou

**Affiliations:** 1 AWS Center for Quantum Computing, McKinney, USA

The manuscript authored by O. Kieler et al. presents the development of a gold stud bump-based flip-chip technology for integrating photodiodes and fibers with superconducting circuits. The authors provide detailed descriptions of the fabrication process, optimize the bump geometry, and evaluate the integrated devices at both room and cryogenic temperatures. They demonstrate a high fabrication yield and offer a thorough characterization of device performance, including an analysis of misalignment effects.

 Although gold bump integration techniques are well-documented in the literature, this manuscript stands out by providing detailed fabrication protocols that could enable other research groups to reproduce their results. This contribution is therefore a valuable addition to the field.

 As many key points have already been addressed in other reviewers' comments and the authors have provided satisfactory responses in the revised manuscript (version 2), I have the following remaining comments and minor suggestions:

1. The authors validate the yield rate by testing DC connections at cryogenic temperatures. Have they conducted any microwave measurements, such as S11 or S21, to assess the stability of the device under high-frequency signals? If the microwave performance remains consistent after multiple cooling cycles, it would further enhance the potential applications of this fabrication process in quantum technologies.
2. The authors attribute the limited coupling efficiency to imperfections in the ferrule-sleeve integration. Given this limitation, what was the rationale behind selecting this method for coupling the fiber to the photodiode? A discussion addressing this choice would provide helpful context for readers.
3. In Figure 7, the statement "They both exhibit a similar behavior" appears inaccurate, as the coupling efficiencies for the two fibers differ significantly.

Overall, the manuscript is clearly written and easy to follow. Once the authors address the questions above, I support the indexing the manuscript.

Is the rationale for developing the new method (or application) clearly explained?

Yes

Is the description of the method technically sound?

Yes

Are the conclusions about the method and its performance adequately supported by the findings presented in the article?

Yes

If any results are presented, are all the source data underlying the results available to ensure full reproducibility?

Yes

Are sufficient details provided to allow replication of the method development and its use by others?

Yes

Reviewer Expertise:

Quantum acoustics, hybrid quantum systems, superconducting circuits, fabrications

I confirm that I have read this submission and believe that I have an appropriate level of expertise to confirm that it is of an acceptable scientific standard, however I have significant reservations, as outlined above.

# Reviewer report: Reviewer response for version 2

**DOI:** 10.21956/openreseurope.20301.r46093

**Published:** 2024-11-23

**Authors:** Jie Ren

**Affiliations:** 1 Shanghai Institute of Microsystem and Information Technology, Chinese Academy of Sciences, Shanghai, China

The author answered all my questions and made some modifications and additions to the manuscript. I believe this can be indexed as a method article.

Is the rationale for developing the new method (or application) clearly explained?

Partly

Is the description of the method technically sound?

Partly

Are the conclusions about the method and its performance adequately supported by the findings presented in the article?

Partly

If any results are presented, are all the source data underlying the results available to ensure full reproducibility?

No source data required

Are sufficient details provided to allow replication of the method development and its use by others?

No

Reviewer Expertise:

SFQ circuit design, low-temperature measurement

I confirm that I have read this submission and believe that I have an appropriate level of expertise to confirm that it is of an acceptable scientific standard.

# Reviewer report: Reviewer response for version 1

**DOI:** 10.21956/openreseurope.18891.r41246

**Published:** 2024-06-27

**Authors:** Jie Ren

**Affiliations:** 1 Shanghai Institute of Microsystem and Information Technology, Chinese Academy of Sciences, Shanghai, China

This article introduces the gold stud bump-based flip-chip technology applied in optical-driven superconducting circuits. The research and development of flip-chip and optical pulse drive technology for superconducting circuits were mainly discussed, with a focus on the process of installing photodiodes and fiber optic sleeves on silicon substrates. And certain verification was carried out in the process of flip soldering. The highly reliable packaging of low-temperature chips is a recent research hotspot. There have been some reports on the packaging of low-temperature superconducting chips using gold bumps (such as 1. R. Das et al., "Interconnect Scheme for Die-to-Die and Die-to-Wafer-Level Heterogeneous Integration for High-Performance Computing," 2019 IEEE 69th Electronic Components and Technology Conference (ECTC), Las Vegas, NV, USA, 2019, pp. 1611-1621, doi: 10.1109/ECTC.2019.00248 and 2. K. Li *et al*., "Golden Bump Based Flip-Chip Interconnection for Superconducting Multi-Chip Module," in *IEEE Transactions on Applied Superconductivity*, vol. 33, no. 5, pp. 1-6, Aug. 2023, Art no. 1200106, doi: 10.1109/TASC.2023.3263132.). The packaging technology demonstrated in this paper have not shown significant advantages and innovations compared to existing works. Therefore, it is recommended to consider whether it is suitable for indexed after major revision. Some specific suggestions are as follows:

 1. Please clearly explain the innovation of the gold-stud-bump-based flip-chip packaging technology in this article.

 2. The article mentions that changing the wire can intuitively obtain the change in the diameter of the protrusion, but by adjusting the protrusion preparation parameters, 25 μ m wire should also be able to achieve a preparation effect of nearly equivalent nail head protrusion diameter for 17 μ m wire. In ref K. Li et al., "Golden Bump Based Flip-Chip Interconnection for Superconducting Multi-Chip Module," in IEEE Transactions on Applied Superconductivity, vol. 33, no. 5, pp. 1-6, Aug. 2023, Art no. 1200106, doi: 10.1109/TASC.2023.3263132., it is possible to achieve a diameter of approximately 2.2 times the diameter of the gold wire. This data is far superior to the results of this article.

 3. The gold bump prepared by the nail head method will retain a certain amount of tail wire, and the gold bump maintains a certain hardness. When the tail wire length is different, will it affect the selection of bonding parameters?

 4. The article mentioned that the test results showed no failure was found after multiple thermal cycles of flip soldering, which means that the yield rate was 100% after both electrical and thermal cycles, but no detailed testing data was provided, please add them.

 5. It can be seen that no phenomenon of open circuit or short circuit under DC testing after the structural interconnection undergoes thermal cycling However, it cannot fully explain whether there are cracks or other effects on the connection interface after low-temperature cycling at the bump, I believe it is necessary to characterize the cross-section at the bump interface to check this.

 6. After flip soldering, the photodiode and fiber optic sleeve of the chip undergo five cooling and preheating cycles between 4 K and 300 K, and the sleeve remains stable. However, the number of 5 cycles is too small. It is recommended to increase the number of experiments. In addition, it is recommended to increase the verification of shear force to quantify the verification effect.

 7. The fiber optic sleeve fixed with Stycast 12661 adhesive did not show any looseness or damage after undergoing temperature cycling, but the CTE of epoxy resin differs significantly from that of other components. Will different expansion and deformation situations cause the sleeve to tilt or shift in alignment, which will affect the subsequent fiber optic connection?

 8. More details should be provided when explaining the results shown in Figure 7. For instance, what is the temperature the measurement carries out? what is the diameter of the ferrules and how is it compared to that of the glass sleeves? And what is the distance between the fiber end and the integrated lens on the backside of the PD?

 9. The unideal results in Figure 7 are attributed to the mechanical imperfections. My question is what kind of mechanical imperfections are causing these and how?

Is the rationale for developing the new method (or application) clearly explained?

Partly

Is the description of the method technically sound?

Partly

Are the conclusions about the method and its performance adequately supported by the findings presented in the article?

Partly

If any results are presented, are all the source data underlying the results available to ensure full reproducibility?

No source data required

Are sufficient details provided to allow replication of the method development and its use by others?

No

Reviewer Expertise:

SFQ circuit design, low-temperature measurement

I confirm that I have read this submission and believe that I have an appropriate level of expertise to state that I do not consider it to be of an acceptable scientific standard, for reasons outlined above.

## References

[1] Interconnect Scheme for Die-to-Die and Die-to-Wafer-Level Heterogeneous Integration for High-Performance Computing. *IEEE* .2019;

[2] Golden Bump Based Flip-Chip Interconnection for Superconducting Multi-Chip Module. *IEEE* .2023;

# Response

**Published:** 2024-10-15

**Authors:** Oliver Kieler

**Affiliations:** Quantum Electronics, Physikalisch-Technische Bundesanstalt, Braunschweig, Lower Saxony, Germany

This article introduces the gold stud bump-based flip-chip technology applied in optical-driven superconducting circuits. The research and development of flip-chip and optical pulse drive technology for superconducting circuits were mainly discussed, with a focus on the process of installing photodiodes and fiber optic sleeves on silicon substrates. And certain verification was carried out in the process of flip soldering. The highly reliable packaging of low-temperature chips is a recent research hotspot. There have been some reports on the packaging of low-temperature superconducting chips using gold bumps (such as 1. R. Das et al., "Interconnect Scheme for Die-to-Die and Die-to-Wafer-Level Heterogeneous Integration for High-Performance Computing," 2019 IEEE 69th Electronic Components and Technology Conference (ECTC), Las Vegas, NV, USA, 2019, pp. 1611-1621, doi: 10.1109/ECTC.2019.00248 and 2. K. Li et al., "Golden Bump Based Flip-Chip Interconnection for Superconducting Multi-Chip Module," in IEEE Transactions on Applied Superconductivity, vol. 33, no. 5, pp. 1-6, Aug. 2023, Art no. 1200106, doi: 10.1109/TASC.2023.3263132.). The packaging technology demonstrated in this paper have not shown significant advantages and innovations compared to existing works. Therefore, it is recommended to consider whether it is suitable for indexed after major revision. Some specific suggestions are as follows:

**Comment: **1. Please clearly explain the innovation of the gold-stud-bump-based flip-chip packaging technology in this article.

**Response: **Please see comment 4 of the second reviewer for an answer and corresponding changes to the manuscript.

**Comment: **2. The article mentions that changing the wire can intuitively obtain the change in the diameter of the protrusion, but by adjusting the protrusion preparation parameters, 25 μ m wire should also be able to achieve a preparation effect of nearly equivalent nail head protrusion diameter for 17 μ m wire. In ref K. Li et al., "Golden Bump Based Flip-Chip Interconnection for Superconducting Multi-Chip Module," in IEEE Transactions on Applied Superconductivity, vol. 33, no. 5, pp. 1-6, Aug. 2023, Art no. 1200106, doi: 10.1109/TASC.2023.3263132., it is possible to achieve a diameter of approximately 2.2 times the diameter of the gold wire.

**Response: **This data is far superior to the results of this article. We thank the reviewer for mentioning this article and agree that it needs to be cited. We added the mentioned reference and extended the sentence at the end of Section 2: “This average bump size is slightly smaller than described in [14] 65…80 µm and slightly larger than in [15] nearly 45 µm.”.

**​​​​​​​Comment: **3. The gold bump prepared by the nail head method will retain a certain amount of tail wire, and the gold bump maintains a certain hardness. When the tail wire length is different, will it affect the selection of bonding parameters?

**Response: **The tail length was optimized to be as short as possible, because a long tail will result in a large effective area of the bump after coining and flip-chip, which will directly increase the probability of shorts between the signal and ground lines of the CPW. Please see comment 1 of the first reviewer for a further answer and corresponding changes to the manuscript.

**​​​​​​​Comment:** 4. The article mentioned that the test results showed no failure was found after multiple thermal cycles of flip soldering, which means that the yield rate was 100% after both electrical and thermal cycles, but no detailed testing data was provided, please add them.

**Response: **To us it is not fully clear what the reviewer exactly means with “testing data” and “electrical ...cycles” (e.g., DC resistance before and after cool down, optical photographs, ...). In this regard we refer to Fig. 4, where we changed the caption by adding the statement: “This photograph was taken before the cool-down. After cooling down there are no visible differences to observe.” Additionally, we like to refer to the next comment and answer, where we mention high-frequency measurements for subsequent cool-downs.

**​​​​​​​Comment: **5. It can be seen that no phenomenon of open circuit or short circuit under DC testing after the structural interconnection undergoes thermal cycling. However, it cannot fully explain whether there are cracks or other effects on the connection interface after low-temperature cycling at the bump, I believe it is necessary to characterize the cross-section at the bump interface to check this.

**Response: **We have removed some PD after flip chip bonding and cool down cycles and inspected the remaining surface of the chip and the PD optically. No indication of cracks could be found. We agree that this optical inspection is different than SEM or even TEM measurements, which are, however, beyond the scope of this manuscript. In addition we have performed high-speed measurements using electro-optic sampling of ultrafast voltage pulses generated by the photodiode  on several samples [ https://doi.org/10.1007/s10762-024-00966-1]. This corresponds to RF testing instead of DC testing. From these measurements it is clear that we remain to have proper high-frequency connections even after multiple cool down cycles. We have added corresponding remarks to Section 3: “While the electrical DC measurements give an indication of the electrical connection they do not fully eliminate the possibility of cracks in the connection interface after cool down and warm up. We have not made any cross section SME image of the connection after thermal cycling, but we have tested the high-frequency connection of the flip-chip-mounted PDs after thermal cycling, which is briefly commented on further below. ” and “We also comment on the electrical connection of the PDs as mentioned in the previous paragraph. We have performed high-speed measurements using electro-optic sampling of ultrafast voltage pulses generated from optical excitation of the PDs using femtosecond lasers [17]. Even after several cool-down and warm-up cycles we always measured the same PD response, proving that we have proper high-frequency flip-chip connections even after several thermal cycles.”.

**​​​​​​​Comment: **6. After flip soldering, the photodiode and fiber optic sleeve of the chip undergo five cooling and preheating cycles between 4 K and 300 K, and the sleeve remains stable. However, the number of 5 cycles is too small. It is recommended to increase the number of experiments. In addition, it is recommended to increase the verification of shear force to quantify the verification effect.

**Response: **We thank the reviewer for this comment. Of course every study can be extended and we agree that the number repetitive cycles is limited. Yet, we believe that even after 5 cycles a certain trend can be obtained. We have therefore left the number of cycles unchanged, but added a corresponding remark to Section 3: “We note that five cooling cycles constitute a limited number from which is it impossible to extract a behaviour for 20 or more cooling cycles. Yes, we believe that even five cooling cycles without any observed problem denote a very positive trend which is worth to be reported.”.

**​​​​​​​Comment: **7. The fiber optic sleeve fixed with Stycast 12661 adhesive did not show any looseness or damage after undergoing temperature cycling, but the CTE of epoxy resin differs significantly from that of other components. Will different expansion and deformation situations cause the sleeve to tilt or shift in alignment, which will affect the subsequent fiber optic connection?

**Response: **We believe that such a situation might happen, in particular, if the adhesive is not applied symmetrically with respect to the cross-section of the sleeve. In our experiments we have not observed any tilt or shift in alignment. We have added a corresponding sentence to the caption of Figure 4.

**​​​​​​​Comment: **8. More details should be provided when explaining the results shown in Figure 7. For instance, what is the temperature the measurement carries out? what is the diameter of the ferrules and how is it compared to that of the glass sleeves? And what is the distance between the fiber end and the integrated lens on the backside of the PD?

**Response: **The measurements were carried out at room temperature. The nominal outer diameter of the ferrule was 1.8 mm with a tolerance of +/- 5 μm, the nominal inner diameter of the sleeve was 1.815 mm. The ferrule end rests directly on the PD substrate. To prevent displacement in the z-direction, the fiber is fixed with light pressure against the PD substrate. This explanation was directly adopted in Section 4. The main point that we want to make with Fig. 7 is that despite the mechanical imperfections, maximum optical coupling can be obtained by adjusting the rotation angle of the chip and/or the fiber at room temperature and keep it fixed during operation at 4 K.

**​​​​​​​Comment: **9. The unideal results in Figure 7 are attributed to the mechanical imperfections. My question is what kind of mechanical imperfections are causing these and how?

 ​​​​​​​ **Response: **Please see comment 5 of the first reviewer for a further answer and corresponding changes to the manuscript.

# Reviewer report: Reviewer response for version 1

**DOI:** 10.21956/openreseurope.18891.r41237

**Published:** 2024-06-26

**Authors:** Saif Wakeel

**Affiliations:** 1 University College Cork, Tyndall National Institute, Cork, Ireland

**Your Report**

 The authors extended the use of flip-chip technology for bonding photodiodes and fiber sleeves. I have following comments/revision request before proceeding to next stage. 

1. Effective thermal management is crucial to prevent overheating, especially in high-power applications. Despite these advantages, the flip-chip process is more complex than wire bonding, requiring precision alignment and careful control of the soldering process. **Reference required..**
2. The reliability of flip-chip assemblies is influenced by factors such as solder joint integrity and underfill material properties. If the flip-chip assembly is aimed for operation at cryogenic temperatures, the challenges further increase, as the thermal expansion of the different materials must be considered. .. **Reference required**
3. **Showing complexity of flip chip packaging doesn't look relevant with the current study. If flip chip is that complex then why authors are using it?**
4. **The second paragraph lists the literature one by one but novelty of paper is not clearly mentioned...**
5. Due to the smaller size of the stud bumps, the risk of short circuits during the flip chip process was avoided completely. A mean size of the bumps of (63.8 ± 3.4) µm was determined by analyzing 60 Au-bumps. **Is there a mathematical relation between the bump size and stud bump size to avoid short circuiting?**
6. To achieve less bump deformation, a bonding force of 4 N or 5 N11–13 for the 5 pads (each with one bump) was used **. Do you think increasing pressure may expect the chances of short circuit as solder can spread more?**
7. **What is the effect of changing temperature on delamination? Or shear strength of device? It could be good to include those. While doing thermal cycling thermo-mechanical stresses are generated which result in delamination/failure of devices? **
8. After optimization of the flip-chip procedure we achieved a yield better than 95% for a total of more than 250 mounted PDs. **Can author comment or make a table with how many devices were successful and how many failures?**
9. Figure 3. Tilted SEM image of a PD mounted onto a high-frequency line on a silicon substrate. **To observe delamination, it could be good to do cross section and show bonded interface. **
10. After five cooling-down and warming-up cycles, the mounted sleeves remained stable and stuck firmly onto the chip. **Do you have any zoom in microscopic image to show this? **
11. Figure 4. Optical photograph of two sleeves mounted using flip-chip technology and glued to the silicon substrate. **Is this image before cool down or after cool down? **
12. When only considering the accuracy of 15 µm and the built-in lens of the PD with a diameter of 100 µm. **Is this alignment accuracy or alignment tolerance in both cases shouldn't it be with plus minus sign if they are in both positive and negative directions?**

Is the rationale for developing the new method (or application) clearly explained?

Partly

Is the description of the method technically sound?

Yes

Are the conclusions about the method and its performance adequately supported by the findings presented in the article?

Yes

If any results are presented, are all the source data underlying the results available to ensure full reproducibility?

Yes

Are sufficient details provided to allow replication of the method development and its use by others?

Partly

Reviewer Expertise:

Photonic packaging, electronic packaging, flip-chip technology, reliability of photonic and electronic devices, wafer-scale assembly or bonding of devices.

I confirm that I have read this submission and believe that I have an appropriate level of expertise to confirm that it is of an acceptable scientific standard, however I have significant reservations, as outlined above.

# Response

**Published:** 2024-10-15

**Authors:** Oliver Kieler

**Affiliations:** Quantum Electronics, Physikalisch-Technische Bundesanstalt, Braunschweig, Lower Saxony, Germany

The authors extended the use of flip-chip technology for bonding photodiodes and fiber sleeves. I have following comments/revision request before proceeding to next stage.

**Reviewer comment:**  Effective thermal management is crucial to prevent overheating, especially in high-power applications. Despite these advantages, the flip-chip process is more complex than wire bonding, requiring precision alignment and careful control of the soldering process.  Reference required.

**Author response: **We agree with the referee and added the following reference to Section 1 after the sentence “Effective thermal management is crucial to prevent overheating, especially in high-power applications.”: Gong, L.; Xu, Y.-P.; Ding, B.; Zhang, Z.-H.; Huang,  Z.-Q.;  Thermal management and structural parameters optimization of MCM-BGA 3D package model, *Int. J. Therm. Sci.*, **2020**, vol. 147, Art. no. 106120, https://doi.org/10.1016/j.ijthermalsci.2019.106120. 2.  

**Reviewer comment: **The reliability of flip-chip assemblies is influenced by factors such as solder joint integrity and underfill material properties. If the flip-chip assembly is aimed for operation at cryogenic temperatures, the challenges further increase, as the thermal expansion of the different materials must be considered. .. Reference required

**Author response: **We agree with the referee and shifted the former reference [12] to the end of the sentence “The reliability of flip-chip assemblies is influenced ... as the thermal expansion of the different materials must be considered.” in Section 1, with the new reference number [5]. Moreover, we added the following reference to Section 1 after the sentence “Despite these advantages, the flip-chip process is more complex than wire bonding, requiring precision alignment and careful control of the soldering process.”: Brinlee, S. and Popelar, S.; A physics-of-failure investigation of flip chip reliability based on lead-free solder fatigue modeling, *J. Microelectron. Electron. Packag.*, **2023**, vol. 20(1): 27–35, https://doi.org/10.4071/imaps.1939648.   

**Reviewer comment: **Showing complexity of flip chip packaging doesn't look relevant with the current study. If flip chip is that complex then why authors are using it?

**Author response: **Using the tiny (350 µm × 350 µm) photodiodes a packaging method other than flip-chip/chip-on-chip/pick-and-place is hardly possible. Due to the backside illuminated PD configuration, the flip-chip is the first and from our point of view probably the only choice. We have added the following sentence to Section 1 to clarify this issue: “We like to note that due to the properties of the PDs, which are discussed in more detail further below, flip-chip mounting of the PDs is the most straightforward way of attaching them to a substrate.”

**Reviewer comment: **The second paragraph lists the literature one by one but novelty of paper is not clearly mentioned...

**Author response: **The novelty of our work is to combine opto-electronics and superconducting quantum electronics for a particular application, which is the pulse-driven Josephson Arbitrary Waveform Synthesizer (JAWS). Our Method Article also demonstrates that the whole procedure can now be performed at PTB. Since these facts are already described in the plain summary and introduction we have only changed the last sentence of Section 1 to emphasize that this manuscript denotes a Method Article in ORE and not a Research Article.

**Reviewer comment: **Due to the smaller size of the stud bumps, the risk of short circuits during the flip chip process was avoided completely. A mean size of the bumps of (63.8 ± 3.4) µm was determined by analyzing 60 Au-bumps. Is there a mathematical relation between the bump size and stud bump size to avoid short circuiting?

**Author response: **No equation was derived, but the combination of position of the bump (related to the center of the PD pad) and the size versus the geometrical dimensions of the CPW (line width and gap between ground and center line) will determine the probability of shorts. We have added the following explanation to Section 2: “... for the given dimensions of the three lower contact pads shown in Fig. 1. Here, the important dimensions are the width of the center contact pad (73 µm) and the gap between the center contact pad and the contact pads on both sides (30 µm). Obviously, a decrease of these dimensions, increases the risk of shorts.”.

**Reviewer comment: **To achieve less bump deformation, a bonding force of 4 N or 5 N 11–13 for the 5 pads (each with one bump) was used. Do you think increasing pressure may expect the chances of short circuit as solder can spread more?

**Author response: **Using higher bond force increased the squeezing of the Au bumps and consequently the diameter of the Au bump was increased too. This directly increased the occurrence of shorts, too. In the manuscript this issue has already been addressed, see comment 1 of the first referee and our corresponding answer.

**Reviewer comment: **What is the effect of changing temperature on delamination? Or shear strength of device? It could be good to include those. While doing thermal cycling thermo-mechanical stresses are generated which result in delamination/failure of devices?

**Author response: **No critical delamination/failure due to mechanical stress occurs during thermal cycling of the devices, because between the PD and the carrier chip only Au interfaces are used: Au-bond pads on both chips and Au-bumps. This explanation was directly added to Section 3.  

**Reviewer comment: **After optimization of the flip-chip procedure we achieved a yield better than 95% for a total of more than 250 mounted PDs. Can author comment or make a table with how many devices were successful and how many failures?

**Author response: **95% out of 250 PDs yields approximately 13 PD which were not working. Typically, we mount 2 PDs on each device. In the worst case, i.e., from 125 devices only 13 devices were not fully functional. A device with one working PD is still operational, but in combination with the JAWS chip, only a unipolar waveform can be synthesized. This explanation was directly added to Section 3. 

**Reviewer comment: **Figure 3. Tilted SEM image of a PD mounted onto a high-frequency line on a silicon substrate. To observe delamination, it could be good to do cross section and show bonded interface.

**Author response: **A cross section image would be indeed good, but this was not performed. We did some preliminary mechanical stress test to remove the sleeves and PDs from the carrier chip. We have added the following sentence to Section 3: “No sleeve or PDs dropped off without introducing some external force.” 

**Reviewer comment: ** After five cooling-down and warming-up cycles, the mounted sleeves remained stable and stuck firmly onto the chip. Do you have any zoom in microscopic image to show this?

**Author response: **Unfortunately, we do not have a zoomed image. Yet, we note that after cooling down there are no visible differences to observe. We have added this explanation to the caption of Figure 4. 

**Reviewer comment: **Figure 4. Optical photograph of two sleeves mounted using flip-chip technology and glued to the silicon substrate. Is this image before cool down or after cool down? This photograph was taken before the cool-down.

**Author response: **After cooling down there are no visible differences to observe. We have added this explanation to the caption of Figure 4.  13.  

**Reviewer comment: **When only considering the accuracy of 15 µm and the built-in lens of the PD with a diameter of 100 µm. Is this alignment accuracy or alignment tolerance in both cases shouldn't it be with plus minus sign if they are in both positive and negative directions?

**Author response: **We thank the reviewer for this remark and agree that our wording is not very clear and should be improved. From Fig. 5(b) we extract a variation of the center of the alignment sleeve being less than 15 µm in each direction. Since we have no specific knowledge about the distribution function of this variation for different cross sections and images, we assume a uniform distribution according to Section 4.3.7 of Ref. [18]. This yields a standard uncertainty of the sleeve alignment procedure of 15 µm/sqrt(3) = 9 µm. Please note that according to Ref. [18] it is not necessary to add a +/- sign when stating standard uncertainties. In the manuscript we have added the following text to Section 3: “An intensity analysis of the images (see Figure 5b) allowed us to quantitatively determine the standard uncertainty of the alignment procedure. To this end, we extracted the measured width of both alignment circles shown in Fig. 5(b) and found that the widths differ by less than 15 μm (in each direction). Since we have no specific knowledge about the distribution function for different cross sections and different images, we assume an uniform distribution according to Ref. [18]. This yields a standard uncertainty of the alignment procedure of 15 µm/ *3*  = 9 µm.”. Moreover, we have replaced “accuracy” by “uncertainty” at several places throughout the manuscript and adopted the expression for the standard uncertainty of the sleeve alignment procedure as explained above to be 9 µm. Finally, Ref. [18] has been added.

# Reviewer report: Reviewer response for version 1

**DOI:** 10.21956/openreseurope.18891.r40361

**Published:** 2024-06-11

**Authors:** Zhi Ting Ye

**Affiliations:** 1 National Chung Cheng University, Chiayi, Taiwan

The paper proposed the size of the gold stud bumps, and flip-chip parameters were optimized, and the fiber sleeves were aligned with specially fabricated alignment circles before applying thermocompression with the flip-chip machine.  This is interesting research and the writing is smooth.  The author please respond to the following recommendations:

 Q1. How were the size of the gold stud bumps and flip-chip parameters optimized to increase the yield and success rate of the flip-chip procedure?

 Q2. What were the challenges faced in connecting optical fibers to the photodiodes in an optimal position, and how were they overcome?

 Q3. What were the results of testing the mounted photodiodes at both room temperature and cryogenic temperature and how did mechanical imperfections affect the alignment accuracy?

 Q4. How does the measured photocurrent of the photodiode under irradiation by CW laser light vary with the x-y position of the ferrule over the PD, as shown in Figure 6, and what does this variation indicate about the optical coupling efficiency in the system?

 Q5. In Figure 7, under continuous laser irradiation, how does the DC output current of the photodiode change with the rotation angle of the chip relative to the ferrule? What impact does this change have on the alignment accuracy and optical coupling efficiency in the system?

 Also, please explain the reason why the green line trend is asymmetric.

Is the rationale for developing the new method (or application) clearly explained?

Yes

Is the description of the method technically sound?

Yes

Are the conclusions about the method and its performance adequately supported by the findings presented in the article?

Yes

If any results are presented, are all the source data underlying the results available to ensure full reproducibility?

Yes

Are sufficient details provided to allow replication of the method development and its use by others?

Yes

Reviewer Expertise:

high power packaged

I confirm that I have read this submission and believe that I have an appropriate level of expertise to confirm that it is of an acceptable scientific standard, however I have significant reservations, as outlined above.

# Response

**Published:** 2024-10-15

**Authors:** Oliver Kieler

**Affiliations:** Quantum Electronics, Physikalisch-Technische Bundesanstalt, Braunschweig, Lower Saxony, Germany

We appreciate the referees’ efforts made to review our manuscript entitled “Development of flip-chip technology for the optical drive of superconducting circuits”, which we submitted as Method Article to Open Research Europe. In the revised manuscript, we have taken care of the requests and suggestions made by the referees, which we found very helpful to improve our Method Article. The corresponding changes have been highlighted as **green text** in the revised manuscript. In the following we provide a point-to-point reply to the referees’ comments. For each single point we first provide an answer to the referee and then explain the corresponding changes to the manuscript (if there are any). **Reviewer 1: ** The paper proposed the size of the gold stud bumps, and flip-chip parameters were optimized, and the fiber sleeves were aligned with specially fabricated alignment circles before applying thermocompression with the flip-chip machine.  This is interesting research and the writing is smooth.  The author please respond to the following recommendations:

**Q1. How were the size of the gold stud bumps and flip-chip parameters optimized to increase the yield and success rate of the flip-chip procedure?**

 For optimizing the bond parameters for making the stud bumps, the most significant parameters were slightly varied. It was then checked, whether the diameter of the balls increases or decreases. We finally chose those parameters, where the balls had the smallest diameter and simultaneously “long” tails were avoided. In this way we found the following parameters: ultra-sonic power 100 mW, Electronic Flame-Off (EFO) power 90%, bond-time 150 ms, bond-power 12 cN, chuck-temperature 40°C, tool-temperature 160°C. We added the following sentence to paragraph 2 of Section 2: “The optimal bond parameters were determined by slightly varying each individual parameter and checking the diameter of the gold stud bumps.”. For optimizing the flip-chip bonding process, the most significant parameters were slightly varied again. The change of the size of the stud bumps after the thermocompression bonding was investigated. We chose those parameters, where the size of the stud bumps  increased the least. To reach this goal, we reduced the bonding force to rather low values of 4 N or 5 N. In this way we found the parameters given in the paper. We added the following sentence to Section 3: “This bonding force range was selected based on prior optimization experiments, where various bonding forces were tested, and it was observed that forces between 4 N and 5 N resulted in the least deformation of the stud bumps, ensuring reliable bonding without compromising the integrity of the photodiode or the carrier chip and without causing short circuits.”.

**Q2. What were the challenges faced in connecting optical fibers to the photodiodes in an optimal position, and how were they overcome?**

 One of the main challenges in connecting optical fibers to the photodiodes optimally is to precisely fix the fiber's position to ensure optimal signal coupling. To address this, the glass body of the pigtail ferrule is inserted into a glass sleeve that has been carefully glued directly above the photodiode (PD). This method effectively fixes the fiber's position in the XY plane. However, challenges remain in fine-tuning the rotational angle (θ) and the Z-position. At room temperature, and under continuous wave (CW) light, the optimal angle is determined experimentally. Once identified, the fiber is lightly pressed onto the backside of the PD using a fixture, ensuring the alignment is secure. This method prevents an air gap forming between the PD and the glass ferrule after cooling and ensures that the angle remains correct. By handling these aspects carefully, an optimal connection is achieved that maintains alignment and minimizes signal loss both at room temperature and after cooling to 4 K. Since the above explanation is already given in the text, we have left the manuscript unchanged with respect to this point.

**Q3. What were the results of testing the mounted photodiodes at both room temperature and cryogenic temperature and how did mechanical imperfections affect the alignment accuracy?**

 When the fibers were aligned, mounted, and fixed at room temperature (as described in Q2), the coupling efficiency did not deteriorate after cooling down to liquid helium temperatures. Mechanical imperfections at the fiber end only affected the optimal rotational angle (θ) but did not impact the XY positioning accuracy of the fiber relative to the photodiode. This is because the XY alignment is determined by the precise positioning of the glass sleeve over the photodiode. Thus, the careful initial alignment and mounting ensured stable performance even under cryogenic conditions, with mechanical imperfections playing a minimal role. We changed the following sentence in Section 4: “Thus, it is clear that mechanical imperfections of the ferrule-sleeve combination limit the overall coupling efficiency.”.

**Q4. How does the measured photocurrent of the photodiode under irradiation by CW laser light vary with the x-y position of the ferrule over the PD, as shown in Figure 6, and what does this variation indicate about the optical coupling efficiency in the system?**

 The photocurrent becomes smaller when you leave the focus range of the PD lens, as an increasing proportion of the incident light is not focused on the PD. The range in which this effect occurs can be derived from the data, which also plays a role in the required adjustment accuracy of the sleeve in relation to the center of the lens. Optimum coupling is achieved with an adjustment accuracy of approx. +/- 30 µm in the xy direction in relation to the center of the PD lens, provided that the ferrule end lies planar on the PD lens. We added the following sentence to Section 4: “As the ferrule moves away from the optimal alignment position, the photocurrent decreases, indicating reduced optical coupling efficiency.”.

**Q5. In Figure 7, under continuous laser irradiation, how does the DC output current of the photodiode change with the rotation angle of the chip relative to the ferrule? What impact does this change have on the alignment accuracy and optical coupling efficiency in the system?**

 Also, please explain the reason why the green line trend is asymmetric. Mechanical imperfections (e.g. scratches in the lense, non-planar contact between ferrule and lens, imperfect radiation pattern of the ferrule, deviation within the manufacturer's tolerance of the outer diameter of the ferrule and/or the inner diameter of the sleeve along its main axes, non-cylindrical shapes of the outer diameter of the sleeve and/or the ferrule) affect the optical coupling efficiency. For a given alignment offset between the fiber core and the center of the lens, the transition area of the light from the ferrule end to the lens can be slightly changed by rotating the fiber so that the light coupling into the lens is optimized despite the existence of mechanical imperfections (e.g. scratches in the lens). Since mechanical imperfections are almost unavoidable, the correct angle between the ferrule and lens must be set according to the maximum photocurrent obtained from Figure 7. The behaviour of the green curve is asymmetric due to the different mechanical properties of the employed ferrule as compared to the ferrule used for measuring the red curve. We added the following sentences to Section 4: “This variation is due to mechanical imperfections, such as scratches on the lens, non-planar contact between the ferrule and lens, imperfect radiation patterns of the ferrule, deviations within the manufacturer's tolerance of the outer diameter of the sleeve and/or the inner diameter of the ferrule along its main axes, and non-cylindrical shapes of the outer diameter of the sleeve and/or the ferrule, which affect the optical coupling efficiency. For a given alignment offset, rotating the fiber slightly adjusts the transition area of light between the ferrule end and the lens, allowing light to couple better to the PD’s active area despite these imperfections.”
